# Ultrasonic Array for Obstacle Detection Based on CDMA with Kasami Codes

**DOI:** 10.3390/s111211464

**Published:** 2011-12-02

**Authors:** Cristina Diego, Álvaro Hernández, Ana Jiménez, Fernando J. Álvarez, Rebeca Sanz, Joaquín Aparicio

**Affiliations:** 1 Electronics Department, University of Alcalá de Henares, Campus Universitario. Ctra. Madrid-Barcelona Km. 33.600, Madrid 28805, Spain; E-Mails: alvaro@depeca.uah.es (A.H.); ajimenez@depeca.uah.es (A.J.); rebeca.sanz@depeca.uah.es (R.S.); joaquin.aparicio@depeca.uah.es (J.A.); 2 Electrical Engineering Electronics and Automatics Department, University of Extremadura, Avda. de Elvas s/n, Badajoz 06071, Spain; E-Mail: fafranco@unex.es

**Keywords:** ultrasound, Phased Array, CDMA, B-Scan images

## Abstract

This paper raises the design of an ultrasonic array for obstacle detection based on Phased Array (PA) techniques, which steers the acoustic beam through the environment by electronics rather than mechanical means. The transmission of every element in the array has been encoded, according to Code Division for Multiple Access (CDMA), which allows multiple beams to be transmitted simultaneously. All these features together enable a parallel scanning system which does not only improve the image rate but also achieves longer inspection distances in comparison with conventional PA techniques.

## 1. Introduction

In comparison with ordinary ultrasonic single-element transducers, Phased Arrays (PA) transducers provide a beam steering method without manual or mechanical scanner requirements. PA systems also offer a flexible way to reshape the beam pattern by changing the geometric parameters of the array. On the other hand, single-element transducers can only change beam shape through changes in the excitation, since higher frequency excitations produce narrower beams. Therefore single-element conventional transducers have a limited fieldwork.

Ultrasonic Phased Array techniques are an attractive method to show ultrasound imaging, successfully developed in medical applications. Currently PA systems are applied in cardiac and abdominal diagnoses, where they have proven to be diagnostically useful and have enjoyed commercial success [1]. Besides, ultrasonic test instruments have been widespread also in industrial applications, more specifically *NDT* (Non-Destructive Testing) for more than sixty years [2]. Common features of these methods are:

- Both technologies are used in immersion or in contact with high-density mediums such as human tissues or steel, where the sound propagates with higher speed and less attenuation when compared to airborne transmission.
- Array elements are driven by pulses implying a drop of *SNR* (Signal to Noise Ratio). Therefore these techniques are used in applications where the range of interest is in centimeters rather than for long-distance inspection.
- A remarkable disadvantage of PA systems is their low image rate, because a different emission is required for each image line or angular sector into which the scanned environment is divided.

As a solution for the low image rate, different imaging techniques, such as Synthetic Aperture techniques (SA) [3], are developed in line with PA. In these Synthetic Aperture systems the image rate depends on how the signals are acquired. Hence the time needed to generate the whole image does not depend on the number of scanned sectors but on the array size. Consequently, if the number of sectors is higher than the number of array elements, the image rate of SA systems increases. However, the amount of energy emitted to the environment decreases significantly since the SA system does not transmit with every element in the array simultaneously. Likewise if the number of receivers is lower, the number of combined signals to obtain the whole final image is lower than with PA systems.

Currently *CDMA* (Code Division for Multiple Access) techniques are proliferating in multiuser ultrasonic applications since the encoding of ultrasonic transmission has been already proposed in numerous previous works [4–6]. In these studies different sequences and codes have been considered, such as Gold, Kasami codes or Complementary Sets of Sequences (CSS). In all these encoding schemes, the simultaneous emission and reception from different users is assumed, since every user has its own pseudo orthogonal code assigned univocally identifying it.

In the current work a combination of PA and coded excitation is considered. Coded excitation in medical ultrasound and NDT has been used to improve the signal to noise ratio (*SNR*) without increasing the excitation voltage [7,8]. Other applications of encoded excitation include increasing the frame rate and resolution [9], or spatial resolution and image contrast [10]. To our knowledge, the novel proposal of this work lies in simultaneously steering the beam at different azimuthal angles by emitting different Kasami codes at the same time, one for each image line or sector into which the scanned environment is divided. Transmitting more than one signal simultaneously is usually precluded by the resulting interference between echoes. Nonetheless, thanks to the encoded signal properties, the sector from which the echo is received can be discriminated. Hence, not only higher image rates are achieved, but also *SNR* increases, allowing longer-distance inspection when compared to conventional PA even in airborne transmission.

The manuscript, which is an extended version of the work submitted at SAAEI 2011 (*Annual Seminar on Automatics, Industrial Electronics and Instrumentation*) [11], is organized as follows. In Section 2 the array design is presented. Section 3 shows the proposed encoded transmission system to obtain ultrasound images of the environment. In Section 4 some simulation results are provided. Finally, conclusions are outlined in Section 5.

## 2. Array Design

The simulation environment used to design the array parameters is the Field II program [12,13], specifically designed for PA and ultrasound imaging simulation. The program calculates the acoustic field in certain space positions chosen by the user with the array located at the origin of the coordinate system. In addition to calculating the response of the array, the software allows to obtain the echo generated by punctual reflectors located in the array inspection plane, taking into account spherical divergence and airborne absorption phenomenon.

**Figure 1.**
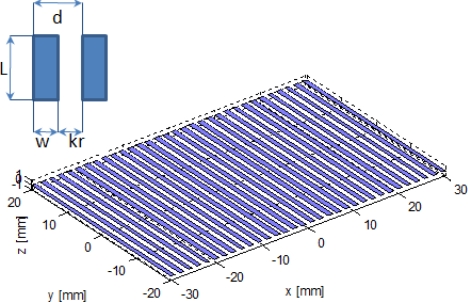
Proposed array design.

**Figure 2.**
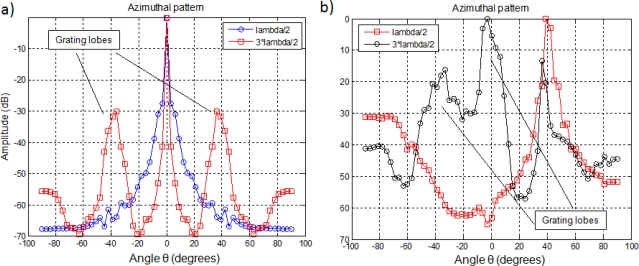
Azimuthal pattern depending in deflection angle *θ*: **(a)** *θ* = 0° and **(b)** *θ* = 40°.

The proposed linear array and its main design parameters are shown in Figure 1, where *L* = 4 cm is the element height; *k_r_* = 1 mm is the inter-element spacing; *w* = 1.16 mm is the individual element width; and *d* = *k_r_* + *w* is known as the pitch. The size of the pitch is $d=\frac{\lambda }{2}$ in order to avoid grating lobes, which constitute peaks in the array azimuthal pattern at angles differing from the main beam orientation. Since an echo imaging system should be sensitive only to targets positioned along the direction of the main beam, these grating lobe peaks reduce the dynamic range for unambiguous imaging. Figure 2(a) shows how the grating lobes increase when the pitch is higher than $\frac{\lambda }{2}$. Besides Figure 2(b) describes how in a $d=\frac{3\cdot \lambda }{2}$ configuration, grating lobes become even bigger than the main lobe as the steered angle increases. In both cases the number *N* of elements in the array is 32.

**Figure 3.**
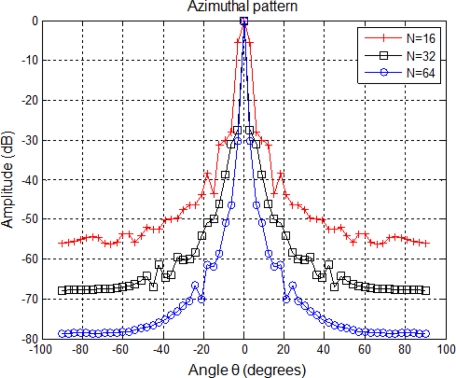
Variation of the azimuth pattern according to the number *N* of elements.

The number *N* of elements in the array does not only determine the width of the beam, as can be observed in Figure 3, but also the amount of emitted energy. In the proposed array, the *N* parameter is equal to 32 elements, which has been chosen as a trade-off between beam width and computational cost.

**Figure 4.**
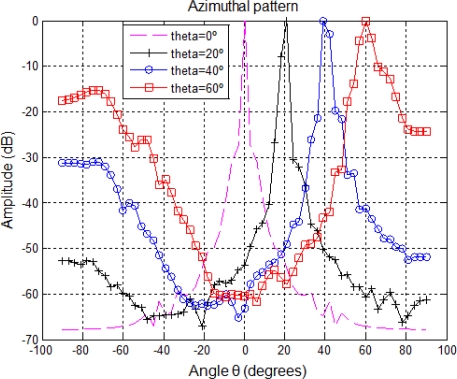
Array azimuth pattern as the steered angle increases *θ* for *N* = 32 elements.

Figure 4 shows the azimuthal pattern of the considered array. It can be observed how the grating lobes increase with the steered angle *θ*, and the main lobe becomes wider. This implies that as the steered angle *θ* moves away from 0°, the image lateral resolution decreases.

## 3. Encoded PA Proposal

**Figure 5.**
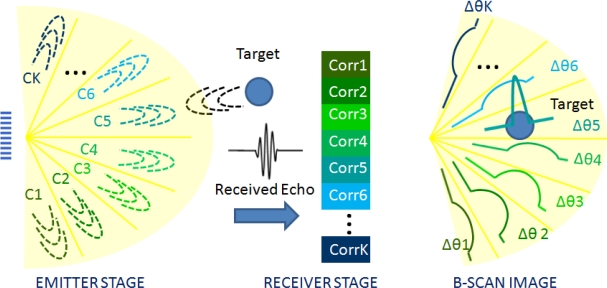
Encoded PA proposal scheme.

Ultrasound images are obtained by steering the beam at different azimuthal angles or sectors Δ*θ*; in order to explore the surrounding environment detecting reflectors. This work proposes the inspection of the whole environment with a single emission based on encoded signals. With this aim, the environment has been divided into *K* angular sectors Δ*θ*_*i*=1..*K*_ and a different code *c_i_* has been assigned to each of these sectors. Hence, as is depicted in Figure 5, the angular sector Δ*θ*_*i*_, from which the echo is received, could be discriminated after a correlation process at the detection stage. Besides, with a single emission the whole environment could be scanned and, thus, not only the image rate but also the SNR is increased.

The effectiveness of CDMA techniques strongly depends on the used codes features. These codes should provide low cross-correlation (CC) values in order to avoid mutual interferences among different sectors. Besides, the codes should have a high auto-correlation peak, in order to distinguish them from noise. In the current work, Kasami codes [14,15], are used over Gold, CSS and LS codes because if the same length and number of pseudo orthogonal codes are considered, they posses a lower correlation bound according to [16].

The transmission proposal consists in a different pseudo orthogonal Kasami code assigned to each angular sector Δ*θ*. The aperiodic auto-correlation function (AACF) of these codes presents small sidelobes that facilitate the detection of the targets. Moreover, it is possible to find a significant number of codes with low values of CC between them. This property allows to steer the beam simultaneously in every sector Δ*θ*_*i*=1..*K*_ with minimum interference between them.

The number *K* of pseudo orthogonal codes depends on their length: if the length *L_c_* increases, so does the number of pseudo orthogonal codes available. There are *K* = 8 pseudo orthogonal Kasami codes with *L_c_* = 63 bits, *K* = 16 codes with *L_c_* = 255 bits, *K* = 32 codes of *L_c_* = 1, 023 bits and so on. The number *K* of available pseudo orthogonal codes determines the number *K* of angular sectors, which is strongly related to the image resolution. Therefore the higher the number *K* of available pseudo orthogonal codes, the higher the image resolution that can be achieved. In this proposal *K* = 32 Kasami codes of *L_c_* = 1, 023 bits are emitted. Longer Kasami codes could have been used, but longer codes imply longer time emission and, for that reason, *L_c_* = 1, 023 bits Kasami codes have been chosen as a trade-off.

**Figure 6.**
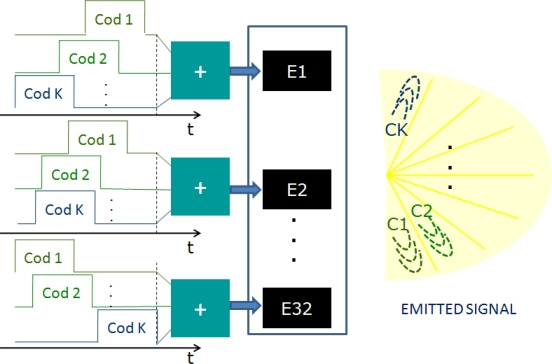
Detail of the emission stage: Each element is driven by the sum of the *K* delayed codes.

Each Kasami code *c_i_* must be emitted by all array elements *E*_1_...*E*_32_, each one with its own delays in order to steer the beam along the azimuthal sector Δ*θ*_*i*_ assigned to the corresponding Kasami code *c_i_*. This process must be carried out for each Kasami code *c*_*i*=1..*K*_ to scan the whole environment. However, taking advantage of the Kasami CC properties, all codes are simultaneously emitted. Therefore, as is shown in Figure 6, every array element is driven by the sum of all Kasami codes *c*_*i*=1..*K*_ with their corresponding delays. Hence, it is possible to scan the whole environment with a single emission. Thanks to the encoded signals, the amount of emitted energy on each azimuthal sector is increased when compared to conventional PA, allowing longer distances inspection.

**Figure 7.**
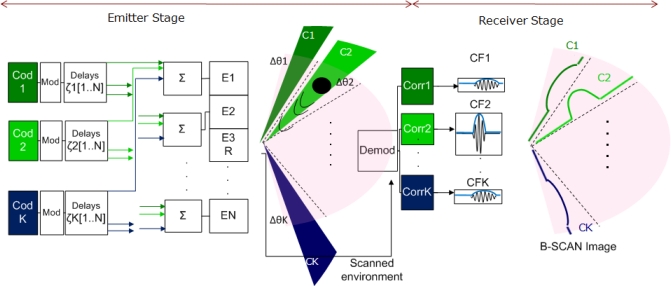
Full system scheme when a reflector is placed in angular sector Δ*θ*_2_.

The block diagram depicted in Figure 7 represents the signal processing of the designed system. The emitter stage is made up of 32 elements, each one of them driven by the sum of *K* = 32 modulated and delayed Kasami codes. Those delays depend on the steered azimuthal sector Δ*θ*_*i*_ assigned to each code *c_i_*. Before the delay stage, Kasami codes are BPSK modulated (Binary Phase-Shift Keying) to focus the energy on the work frequency *f*_0_ = 80 kHz.

On the other hand, the reception stage consists of a single receiver located at the origin of the coordinate system. The echo generated by the reflectors reaches the receiver; where after being correlated with the *K* emitted codes, *K* correlation functions *CF*_*i*=1..*K*_ are obtained. Every correlation function *CF_i_* corresponds to the *A-Scan* signal obtained from every angular sector Δ*θ_i_* that makes up the image. As an example, in Figure 7, if the target is located in sector Δ*θ*_2_, a maximum *CF* will be found when correlating the received echo with *c*_2_. This maximum will provide the reflector location.

## 4. Results and Discussion

Some simulated tests have been carried out in order to validate the proposal. In the following simulations a sector of *S_e_* = 64° is scanned by emitting 32 Kasami codes simultaneously. As explained before, the number *K* of pseudo orthogonal codes available establishes the number of *K* sectors into which the scanned environment is divided, defining thereby the maximum lateral resolution of the image. In this case an environment of *S_e_* = 64° is divided into *K* = 32 angular sectors, implying a maximum lateral resolution $R_{L}=\frac{S_{e}}{K}=2{}^{\circ}$. Also, it must be pointed out that punctual reflectors have been considered.

**Figure 8.**
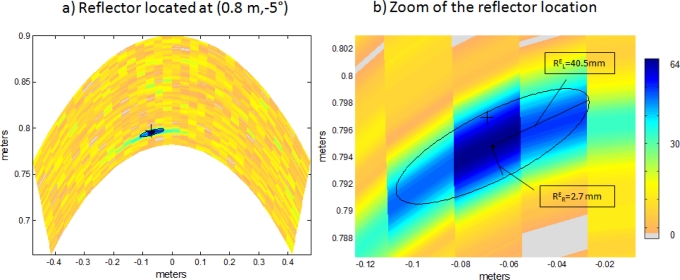
**(a)** Reflector location estimation with Encoded PA after a single simulation. **(b)** Zoom of the reflector location.

**Figure 9.**
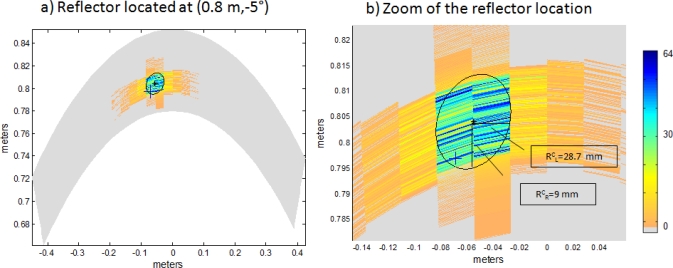
**(a)** Reflector location estimation with Conventional PA after a single simulation. **(b)** Zoom of the reflector location.

In order to show the benefits of the proposed system, a comparison with conventional PA has been carried out. To perform this comparison, the resolution and accuracy of both algorithms are considered. Figures 8(a) and 9(a) show the B-Scan images of the explored environment (*S_e_* = [*−*32°, 32°]), obtained with both algorithms in a noisy environment $\frac{E_{b}}{N_{0}}=10\;\text{dB}$, where *E_b_* is the energy per bit and *N*_0_ is the noise power spectral density. Here an Additive White Gaussian Noise (AWGN) channel is assumed. In both cases, a single punctual reflector is located at polar coordinates (0.8*m,* 0.5°); the real location of this reflector is represented with a cross (+).

Figures 8(b) and 9(b) show a zoom in the B-Scan image where the estimated position of the algorithm is not a single point, but an area whose intensity values are represented by different colors. Intensity values range from 0 to 64, following the trend of previous works [2]. Points whose intensity is higher than a threshold are considered to be in the estimation area, which is delimited by an ellipse and whose center is represented by a star (*). The values of the ellipse axes provide information on lateral *R_L_* and radial *R_R_* resolution achieved; the value of the axis being inversely proportional to the achieved resolution. The threshold considered for both algorithms is half the maximum intensity value.

In Figure 8(b) the major axis of the ellipse provides information on lateral resolution $R\frac{E}{L}=40.5$ mm, whereas the minor axis does on radial resolution $R\frac{E}{R}=2.7$ mm. The same occurs in Figure 9(b), where the major axis of the ellipse provides information on lateral resolution $R_{L}^{C}=28.7$ mm and the minor axis does on radial resolution $R_{R}^{C}=9$ mm. These values are obtained by performing a single simulation.

In order to obtain the average value of lateral $\bar{R_{L}}$ and radial $\bar{R_{R}}$ resolution, a hundred simulations have been carried out for each algorithm. In encoding algorithms the major axis value is $\bar{R_{L}^{E}}=40$ mm which implies lower lateral resolution than conventional PA techniques, whose axis is $\bar{R_{L}^{C}}=30$ mm. It can be observed that in encoded PA algorithm a punctual reflector location is estimated by an area of 3 angular sectors of 2° each. This is due to the bandwidth of the modulated emitted signal which implies a beam widening. Therefore each image sector Δ*θ_i_* is scanned by its assigned code (*c_i_*) and partially by the codes corresponding to its two adjacent sectors *c*_*i*−1_ and *c*_*i*+1_. For that reason, the auto-correlation functions of the adjacent codes provide a correlation maximum too. However, the sector where the reflector is placed provides a sightly higher maximum value and thereby higher intensity than the others, so lateral resolution $\bar{R_{L}^{E}}$ could be improved by merely increasing the threshold. On the other hand, if the threshold is increased, reflectors in longer distances could be discarded.

Radial resolution $\bar{R_{R}^{E}}$ increases in encoded PA techniques due to the characteristics of the auto-correlation function that provides a very sharp maximum. On the other hand, conventional PA techniques emit pulses which in noisy environments are not so robust, providing an ellipse axis of $\bar{R_{R}^{C}}=9.37$ mm whereas encoded algorithms provide $\bar{R_{R}^{E}}=2.6$ mm.

Considering the center of the ellipse (*) as the location estimated by the algorithms and since the real position of the reflector is known (+), the accuracy of both algorithms has been studied. In both cases the error in the position estimation is calculated as the difference between real and estimated position. The average error values of one hundred simulated tests are shown in Table 1, considering separately x-axis and y-axis errors. From these results it can be stated that the accuracy of both algorithms is similar in the x-axis, whereas in the y-axis the error of encoding techniques decreases due to the precision obtained by the auto-correlation function.

**Figure 10.**
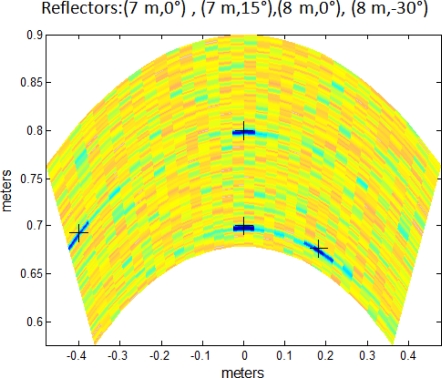
Several reflectors location estimation with Encoded PA.

Moreover, the proposed system can deal with several punctual reflectors as is shown in Figure 10. It can be noticed how the lateral resolution decreases as the steering angle *θ* increases. For that reason, reflectors located at *θ* = −30° have a wider estimation area than reflectors at *θ* = 0°.

Another benefit of the proposed algorithm lies in increasing the image-rate. As explained before, conventional Phased Array techniques need a different emission for each image sector. For every sector a time $t_{\mathrm{sector}}=2\cdot \frac{R_{\max}}{c}$ must be waited for the emitted signal to reach the remotest target and bounce back to the receiver, *R_max_* being the range of this target.

In conventional PA techniques, the required time to generate the whole image is $t_{\mathrm{image}}=t_{\mathrm{emission}}+K\cdot \frac{2\cdot R_{\max}}{c}$. Since the emitted signal is a pulse, the emission time *t_emission_* is negligible, therefore $t_{\mathrm{image}}\approx K\cdot \frac{2\cdot R_{\max}}{c}$. Thus, in ordinary Phased Array systems low image rate is achieved for high resolution images. In the encoded algorithm, *K* beams, steered in *K* different sectors, are emitted at the same time so that the whole environment *S_e_* can be scanned with a single emission. Hence it is only necessary a time $t_{\mathrm{image}}=t_{\mathrm{emission}}+2\cdot \frac{R_{\max}}{c}$ to obtain the B-Scan image.

As an example, if the environment *S_e_* = 64° is divided into *K* = 32 sectors and the range to the remotest target is *R_max_* = 1 m, then the A-Scan time *t_line_* is $t_{\mathrm{line}}=2\cdot \frac{R_{\max}}{c}=5.8$ ms. Conventional PA techniques require *t_image_* = 32 · *t_line_* = 148.59 ms to obtain the B-Scan image. Encoded PA emits Kasami codes of 1023 bits, which implies a *t_emission_* = 25.6 ms. Therefore, it requires a B-Scan time *t_image_* = *t_emission_* + *t_line_* = 26.5 + 5.8 = 31.4 ms which is 5 times faster than PA techniques.

## 5. Conclusions

An ultrasonic sensory array has been defined, with a number of *N* = 32 elements, a pitch *d* = 1.16 mm and a height of *L* = 4 cm. By analyzing the azimuth pattern, it is possible to verify the feasibility of the array for airborne positioning. The transmission of every element in the array has been encoded with a pseudo orthogonal Kasami code for each direction Δ*θ*; at which the beam is steered. Unlike conventional PA techniques, this encoding permits *K* = 32 beams, steered in *K* = 32 different sectors, to be simultaneously transmitted. Thanks to the encoded emission, the *SNR* significantly increases when compared to conventional PA algorithms. Therefore, it is possible to scan distances up to 1*m* even in noisy environments. Furthermore, the image generation rate, which turns out to be an important issue in real-time operation, is thereby increased in comparison with conventional PA systems.

## Figures and Tables

**Table 1.** Algorithms accuracy : Average error in radial and lateral axes after one hundred simulations.

Average Error (mm)	Encoded PA	Conventional PA
X-axis	2.6	2.5
Y-axis	2.2	13.7
